# GPNMB Induces BiP Expression by Enhancing Splicing of *BiP* Pre-mRNA during the Endoplasmic Reticulum Stress Response

**DOI:** 10.1038/s41598-017-11828-3

**Published:** 2017-09-22

**Authors:** Yasuhiro Noda, Kazuhiro Tsuruma, Masafumi Takata, Mitsue Ishisaka, Hirotaka Tanaka, Yusuke Nakano, Yuki Nagahara, Masamitsu Shimazawa, Hideaki Hara

**Affiliations:** Molecular Pharmacology, Department of Biofunctional Evaluation, Gifu Pharmaceutical University, Gifu, Japan

## Abstract

Glycoprotein nonmetastatic melanoma protein B (GPNMB) has a neuroprotective effect against neuronal cell death caused by the accumulation of abnormal mutated proteins. It is known that the accumulation of pathological proteins induces endoplasmic-reticulum (ER) stress leading to cell damage. The aim of this study was to determine the role of GPNMB in the ER stress response. GPNMB was greatly up-regulated by thapsigargin-induced ER stress. Under the ER stress conditions, GPNMB relocated to the nucleus and specifically up-regulated expression of BiP at the mRNA level by promoting the *BiP* pre-mRNA splicing, not through the pathways initiated by the three major transducers of the unfolded protein response: IRE1, PERK, and ATF6. Furthermore, we found that the protein level of BiP and the infarction were increased and attenuated, respectively, in *Gpnmb*-transgenic mice after occlusion of the middle cerebral artery, in comparison with wild-type mice. Thus, our findings indicate that GPNMB enhances the BiP expression by promoting the splicing (thereby preventing cell death caused by ER stress) and could be a therapeutic target in ER stress-related disorders.

## Introduction

Glycoprotein nonmetastatic melanoma protein B (GPNMB), also known as osteoactivin, dendritic cell-heparin integrin ligand, and hematopoietic growth factor-inducible neurokinin-1 type, was initially cloned from poorly metastatic melanoma cells^1^. Normally, GPNMB is localised in several tissues and cells, including skeletal muscle, bone, the hematopoietic system, and immune cells, and performs multiple functions in each tissue. For example, within muscle tissue, GPNMB works as an activator of fibroblasts and thus regulates the degeneration/regeneration of the extracellular matrix in denervated skeletal muscle^2^. Furthermore, GPNMB has been shown to promote differentiation of both osteoblasts^3, 4^ and osteoclasts^5, 6^ and to attenuate T-cell activation^7, 8^. Recently, for the first time, we demonstrated the involvement of GPNMB in neuronal cell death induced by the accumulation of abnormal (mutated) proteins as well as possible usefulness of GPNMB as a novel neuroprotective factor in amyotrophic lateral sclerosis (ALS), which is one of the devastating adult-onset neurodegenerative diseases^9^. Therefore, clarifying the biological function of GPNMB may advance the understanding of the pathogenesis of several diseases.

GPNMB is known as a type I transmembrane protein but is often localised in the perinuclear cytoplasmic regions of different cell types^3, 10^. Recently, GPNMB was detected in the endoplasmic reticulum (ER) and the Golgi apparatus, which are important for protein synthesis, folding, and modifications^3, 11^, suggesting that GPNMB may participate in the protein synthesis/processing in the ER and/or Golgi apparatus. Under physiologically or pharmacologically adverse conditions, perturbations of the ER homeostasis affect protein folding, resulting in accumulation of abnormally unfolded proteins within the ER lumen, thereby causing ‘ER stress’^12^. In response to the stress, protein translation is suppressed first, resulting in down-regulation of new protein synthesis and ensuring prevention of further accumulation of unfolded proteins^13^. Then, molecular chaperone proteins such as 78 kDa glucose-regulated protein (GRP78/BiP) are induced to increase the protein-folding capacity of the ER, leading to attenuation of the ER stress^14^. This system is called the unfolded protein response (UPR). Excessive or long-term ER stress results in cell apoptosis^15, 16^, and the cell fate after ER stress is regulated by a balance between apoptosis and UPR. The cell death *via* ER stress has been implicated in several diseases including brain ischemia^17, 18^, several cancers^19^, diabetes^20^, retinal degenerative diseases^21–23^, and various neurodegenerative diseases such as ALS, Alzheimer’s disease, and Parkinson’s disease^24, 25^. In accord with these findings, we proposed the hypothesis that if GPNMB – which has neuroprotective effects in ALS^9^ – also mediates the protein synthesis/processing, then it functions as a fundamental regulator of the ER stress response. The molecular mechanism of GPNMB’s action, especially in ER stress, remains unclear. Therefore, in the present work, we studied the role of GPNMB in the ER stress response.

## Results

### ER-stress induced Neuronal Cell Death was ameliorated through up-regulation of GPNMB

**Figure 1:**
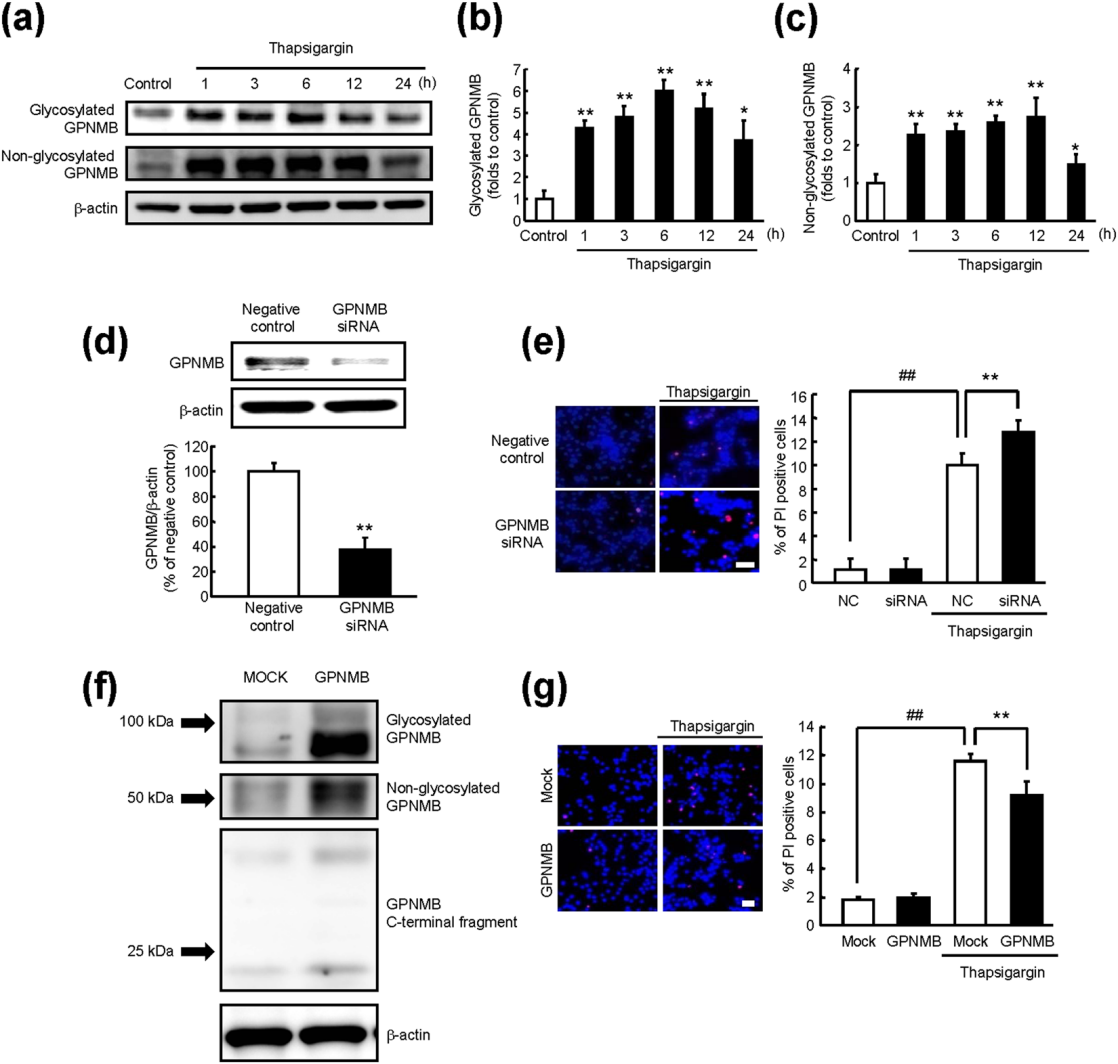
Expression of GPNMB is increased to suppress endoplasmic reticulum (ER) stress-induced cell death. (**a**) Cells were incubated with thapsigargin at 1 μM for the indicated periods. Time course of changes in GPNMB protein levels after thapsigargin treatment was analysed by immunoblotting. (**b**,**c**) The protein levels of glycosylated (**b**) and non-glycosylated (**c**) GPNMB were increased by ER stress. The data are presented as mean ± SEM (*n* = 4). ^*^ *P* < 0.05 and ^**^ *P* < 0.01 compared to control (Dunnett’s test). (**d**) The GPNMB protein levels in cells transfected with anti-*Gpnmb* small interfering RNA (siRNA) or negative control siRNA for 48 h. The data are presented as mean ± SEM (*n* = 3). ^**^ *P* < 0.01 compared to negative control (Student’s *t*-test). (**e**) Representative fluorescence microscopy images showing nuclear staining for Hoechst 33342 (blue) and propidium iodide (PI; red) in cells transfected with an anti-*Gpnmb* siRNA or negative control (NC) 27 h after treatment with 1 μM thapsigargin. The scale bar is 50 μm. The number of cells is shown as the ratio of PI to Hoechst 33342. The data are presented as mean ± SEM (*n* = 4). ^##^ *P* < 0.01 and ^**^ *P* < 0.01 compared to relative controls (Student’s *t*-test). (**f**) The protein levels of GPNMB in cells transfected with the GPNMB-Halo tag vector or empty vector (mock control). (**g**) Representative fluorescence microscopy images showing nuclear staining for Hoechst 33342 (blue) and PI (red) in cells transfected with the GPNMB-Halo tag vector or empty vector (mock control) 27 h after treatment with 1 μM thapsigargin. The scale bar is 50 μm. The number of cells is shown as the ratio of PI to Hoechst 33342. The data are presented as mean ± SEM (*n* = 4). ^##^ *P* < 0.01 and ^**^ *P* < 0.01 compared to relative controls (Student’s *t*-test).

We initially explored the involvement of GPNMB in the ER stress response. The time course of changes in the GPNMB protein levels after thapsigargin-induced ER stress is shown in Fig. 1a. Previously, we confirmed expression of two isoforms of GPNMB in NSC34 cells: glycosylated and non-glycosylated, with molecular weight approximately 100 and 60 kDa, respectively^9^. In the present study, glycosylated and non-glycosylated GPNMB were up-regulated by ER stress and maintained the high expression levels from 1 to 24 h after the thapsigargin treatment (Fig﻿. 1b and c). Furthermore, as shown in Fig. 1e, the anti-*Gpnmb* siRNA aggravated the thapsigargin-induced cell death in comparison with a negative control siRNA. In this condition, the anti-*Gpnmb* siRNA decreased the GPNMB protein levels to 60% of the negative control (Fig. 1d). In case of GPNMB overexpression (Fig. 1f), conversely, GPNMB had a protective effect against the ER stress-induced cell death (Fig. 1g). These findings indicated that expression of the GPNMB protein was regulated by ER stress, and GPNMB influenced the ER stress-induced cell death.

### GPNMB regulates BiP expression

**Figure 2:**
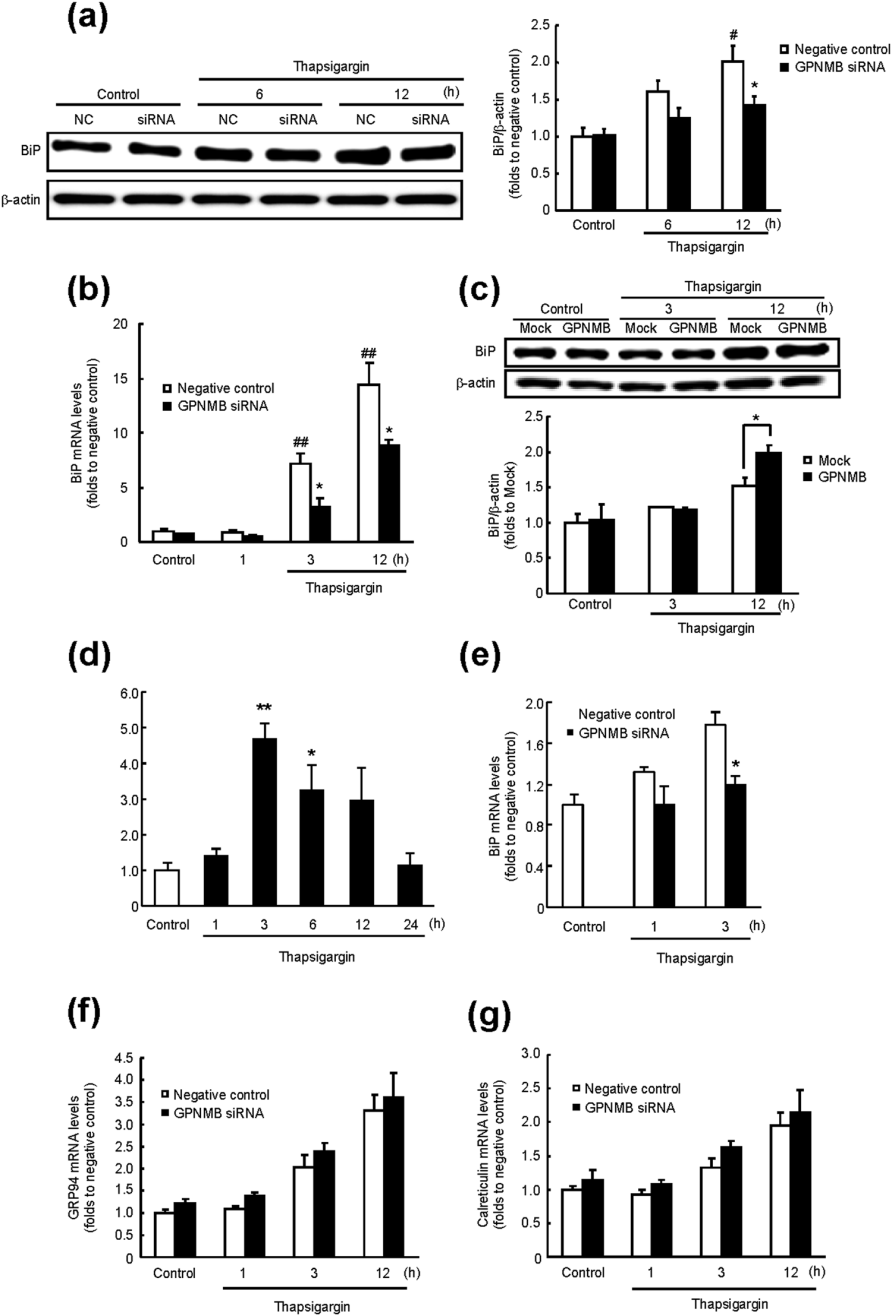
GPNMB regulates *BiP* expression under conditions of endoplasmic reticulum (ER) stress. (**a**) The BiP protein levels were examined by immunoblotting. Cells transfected with anti-*Gpnmb* small interfering RNA (siRNA) or negative control (NC) siRNA were incubated with 1 µM thapsigargin for the indicated periods. The data are presented as mean ± SEM (*n* = 3 to 6). ^#^ *P* < 0.05 compared to control (Dunnett’s test); ^*^ *P* < 0.05 compared to time-matched negative control (Student’s *t*-test). (**b**) The cells that were transfected with anti-*Gpnmb* siRNA or negative control siRNA were incubated with 1 μM thapsigargin for the indicated periods. Expression of *BiP* mRNA was examined using RT-PCR. The data are presented as mean ± SEM (*n* = 4). ^##^ *P* < 0.01 compared to control (Dunnett’s test); ^*^ *P* < 0.05 compared to time-matched negative control (Student’s *t*-test). (**c**) The BiP protein levels were evaluated by immunoblotting. Cells that were transfected with the GPNMB-Halo tag vector or empty vector (mock control) were incubated with 1 μM thapsigargin for the indicated periods. The data are presented as mean ± SEM (*n* = 3 to 5). ^*^ *P* < 0.05 compared to time-matched mock control (Student’s *t*-test). (**d**) HT22 cells were incubated with thapsigargin at 50 nM for the indicated periods. Time course of changes in GPNMB protein levels after thapsigargin treatment was analysed by immunoblotting. The data are presented as mean ± SEM (*n* = 5). ^*^ *P* < 0.05 and ^**^ *P* < 0.01 compared to control (Dunnett’s test). (**e**) HT22 cells transfected with anti-*Gpnmb* siRNA or negative control siRNA were incubated with 50 nM thapsigargin. Expression of *BiP* mRNA was examined using RT-PCR. The data are presented as mean ± SEM (*n* = 4). ^*^ *P* < 0.05 compared to time-matched negative control (Student’s *t*-test). Expression levels of (**f**) *Grp94* and (**g**) *Calreticulin* mRNA were examined using RT-PCR. The data are presented as mean ± SEM (*n* = 4).

ER-resident molecular chaperones such as BiP, glucose-regulated protein 94 (GRP94), and calreticulin are induced by ER stress for the purpose of refolding the unfolded proteins, thus ensuring homeostasis of the ER^26, 27^. Therefore, we next tested whether GPNMB affects the expression levels of these chaperones. The protein and mRNA levels of BiP in the negative control siRNA group were significantly increased in a time-dependent manner by thapsigargin-induced ER stress; however, both phenomena were suppressed by the GPNMB knockdown under the ER stress conditions (Fig. 2a and b). Conversely, expression of the BiP protein in GPNMB-overexpressing cells under ER stress was higher than that in cells transfected with an empty vector (mock control; Fig. 2c). Moreover, GPNMB was upregulated by ER-stress and it regulated the Bip expression in HT22 cells which were derived from murine hippocampal cells (Fig. 2d and e). These results suggest that GPNMB regulates Bip expression in the several types of cell line under the ER-stress condition. On the other hand, the GPNMB knockdown did not affect the mRNA levels of the other chaperones, i.e. GRP94 and calreticulin (Fig. 2f and g), suggesting that BiP might be specifically regulated by GPNMB at the mRNA level under ER stress.

**Figure 3:**
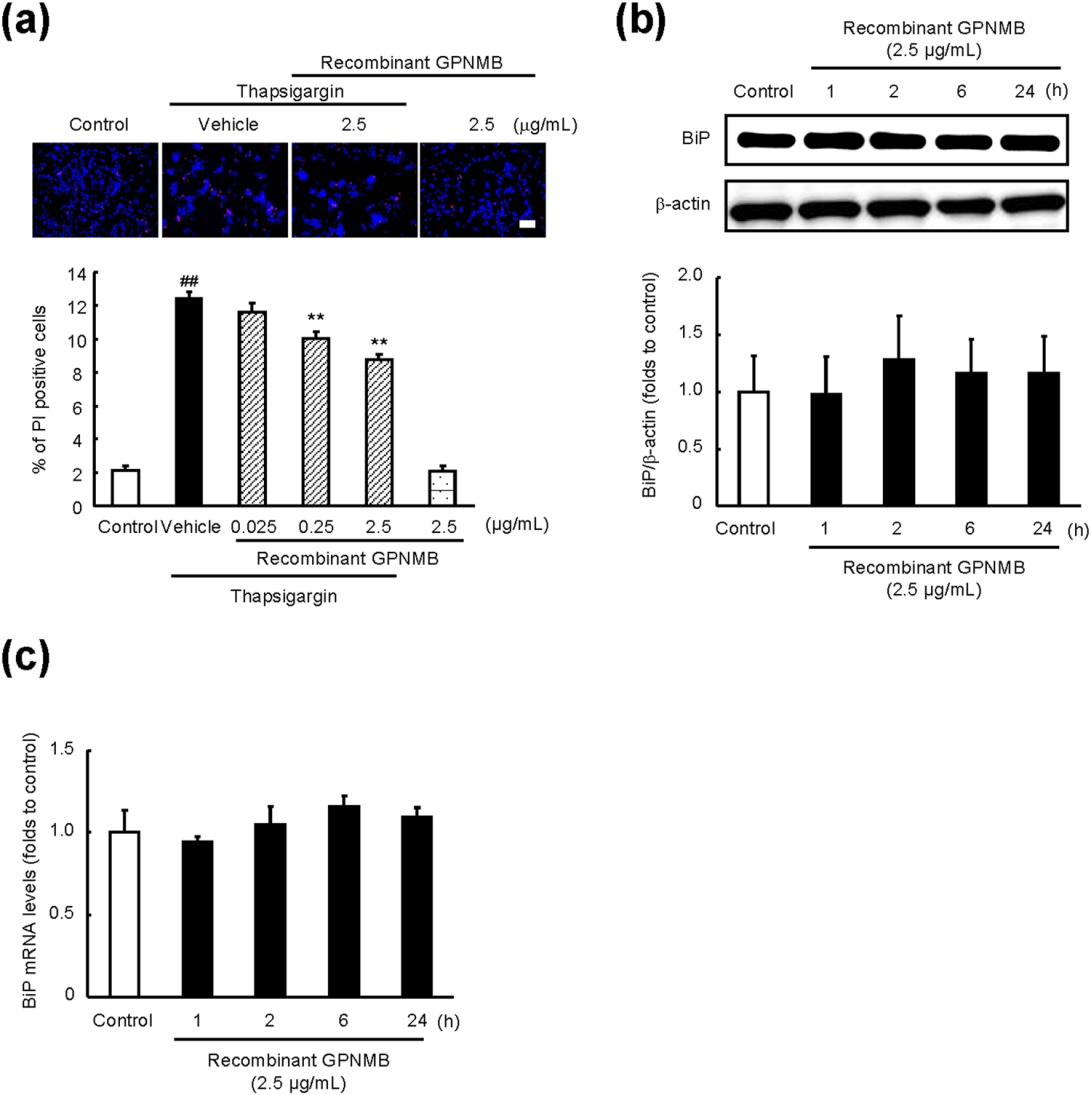
Extracellular fragments of GPNMB do not regulate BiP expression. (**a**) NSC34 cells were incubated with a recombinant extracellular fragment of GPNMB at 0.025–2.5 μg/mL and 1 h later with thapsigargin (1 μM) for 27 h. Representative fluorescence microscopy images showing nuclear staining for Hoechst 33342 (blue) and propidium iodide (PI; red) in NSC34 cells 27 h after thapsigargin treatment. The scale bar is 50 μm. The number of cells is shown as the ratio of PI to Hoechst 33342. The data are presented as mean ± SEM (*n* = 6). ^##^ *P* < 0.01 compared to control (Student’s *t*-test); ^**^ *P* < 0.01 compared to time-matched negative control (Dunnett’s test). (**b**) Time course of changes in BiP protein levels after the cells were incubated with the recombinant GPNMB at 2.5 μg/mL. The BiP protein levels were quantified relative to β-actin. The data are presented as mean ± SEM (*n* = 4). (**c**) Expression of *BiP* mRNA was measured using RT-PCR after incubation of the cells with recombinant GPNMB at 2.5 µg/mL for the indicated periods. The data are presented as mean ± SEM (*n* = 4).

It is known that the extracellular domain of GPNMB is proteolytically cleaved by matrix metalloproteinases, e.g. a disintegrin and metalloproteinase (ADAM) 10 and ADAM12; this process is called ectodomain shedding^28, 29^. Previously, we demonstrated neuroprotection by GPNMB’s extracellular fragments^9^. Nonetheless, in the present study, treatment with a recombinant GPNMB at 2.5 µg/mL did not affect the BiP protein and mRNA expression levels, unless the concentration of GPNMB was high enough to suppress the thapsigargin-induced cell death (Fig. 3a–c). These results indicated that the intracellular part of GPNMB, rather than the extracellular segment, may be important for the induction of BiP.

### GPNMB regulates BiP expression but not *via* UPR pathways

**Figure 4:**
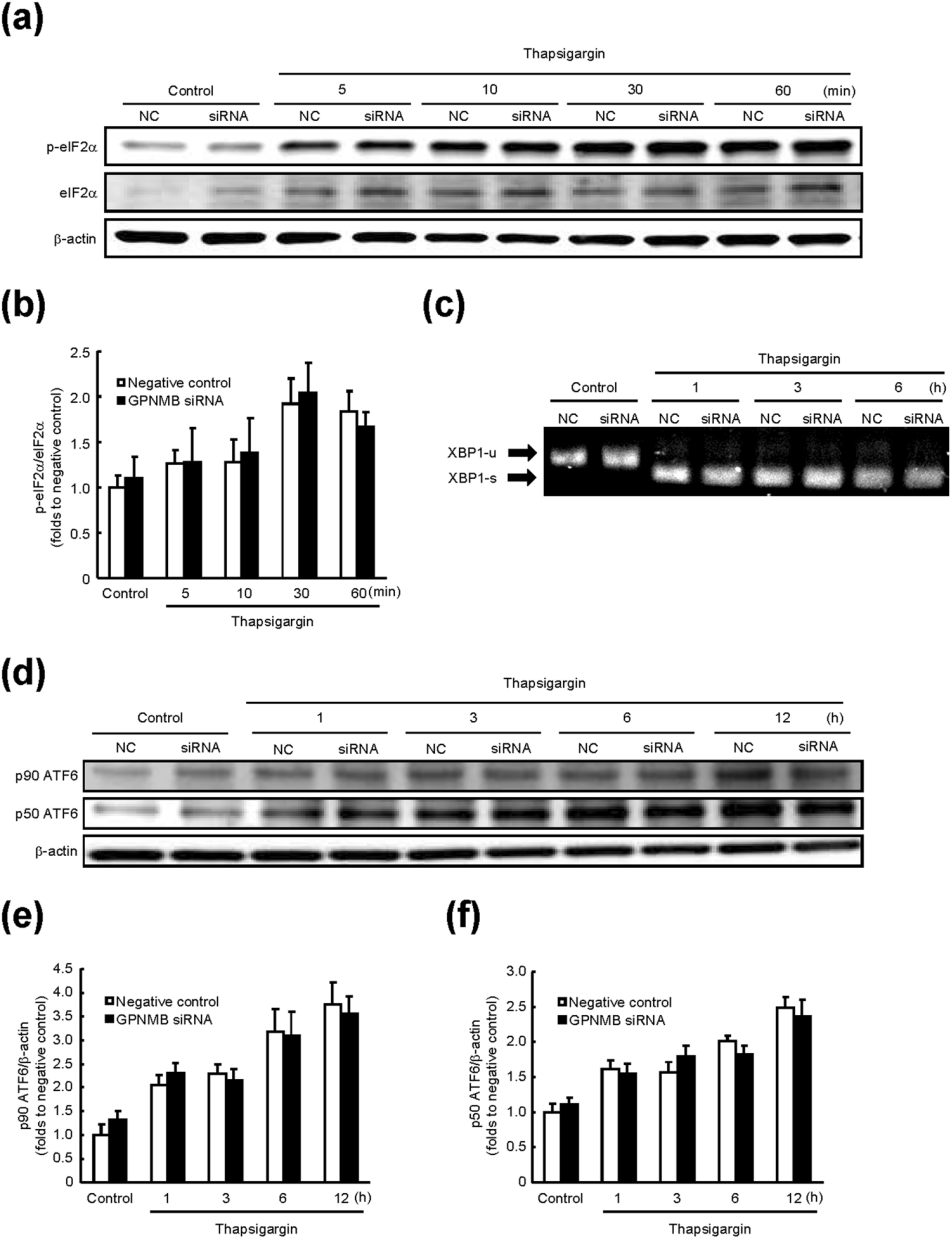
The signal transduction pathways of the unfolding protein response are not regulated by GPNMB. Cells that were transfected with an anti-*Gpnmb* small interfering RNA (siRNA) or negative-control (NC) siRNA were incubated with 1 μM thapsigargin for the indicated periods. (**a**) Time course of the changes in phosphorylated and total eIF2α levels was studied by immunoblotting. (**b**) The protein levels of phosphorylated eIF2α were quantified relative to total eIF2α levels. The data are presented as mean ± SEM (*n* = 4). (**c**) Expression of unspliced (-u) and spliced (-s) *XBP1* mRNA was examined using RT-PCR. (**d**) Time course of changes in ATF6 levels was studied by immunoblotting. (**e**,**f**) Protein levels of p90 and p50 ATF6 were quantified relative to β-actin. The data are presented as mean ± SEM (*n* = 4).

There are three major transducers (IRE1, PERK, and ATF6) that activate the ER stress-response element (ERSE), after transcription of UPR’s target genes^30^. IRE1, a transmembrane kinase, processes the *XBP1* mRNA (unspliced XBP1 mRNA; XBP1-u) to generate mature *XBP1* mRNA (spliced XBP1 mRNA; XBP1-s)^31^. XBP1-s binds directly to ERSE and activates the transcription of BiP. ATF6, a transmembrane transcription factor, is cleaved by site-1 and site-2 proteases under ER stress, and the cleaved ATF6 is translocated to the nucleus to activate the transcription of BiP by binding to ERSE. ATF4, a transcription factor whose translation is up-regulated by the PERK-eIF2 pathway^32^, can activate the *BiP* promoter independently of ERSE. In other words, these three pathways are involved in BiP induction. Therefore, we next tested whether GPNMB regulates BiP expression *via* these three pathways: we measured changes in the key factors that serve as indicators of activation of these pathways. Namely, we evaluated eIF2α phosphorylation, splicing of *XBP1* mRNA, and ATF6 cleavage (Fig. 4). The levels of phosphorylated eIF2α (Fig. 4a and b), XBP1-s (Fig. 4c), and uncleaved (p90) and cleaved (p50) ATF6 (Fig. 4d–f) were statistically significantly increased under ER stress. Significant changes in these factors were not observed between the GPNMB knockdown cells and negative control cells (Fig. 4), indicating that there was no relation between the BiP expression (regulated by GPNMB) and activation of the UPR pathways.

### GPNMB regulates the *BiP* mRNA splicing

**Figure 5:**
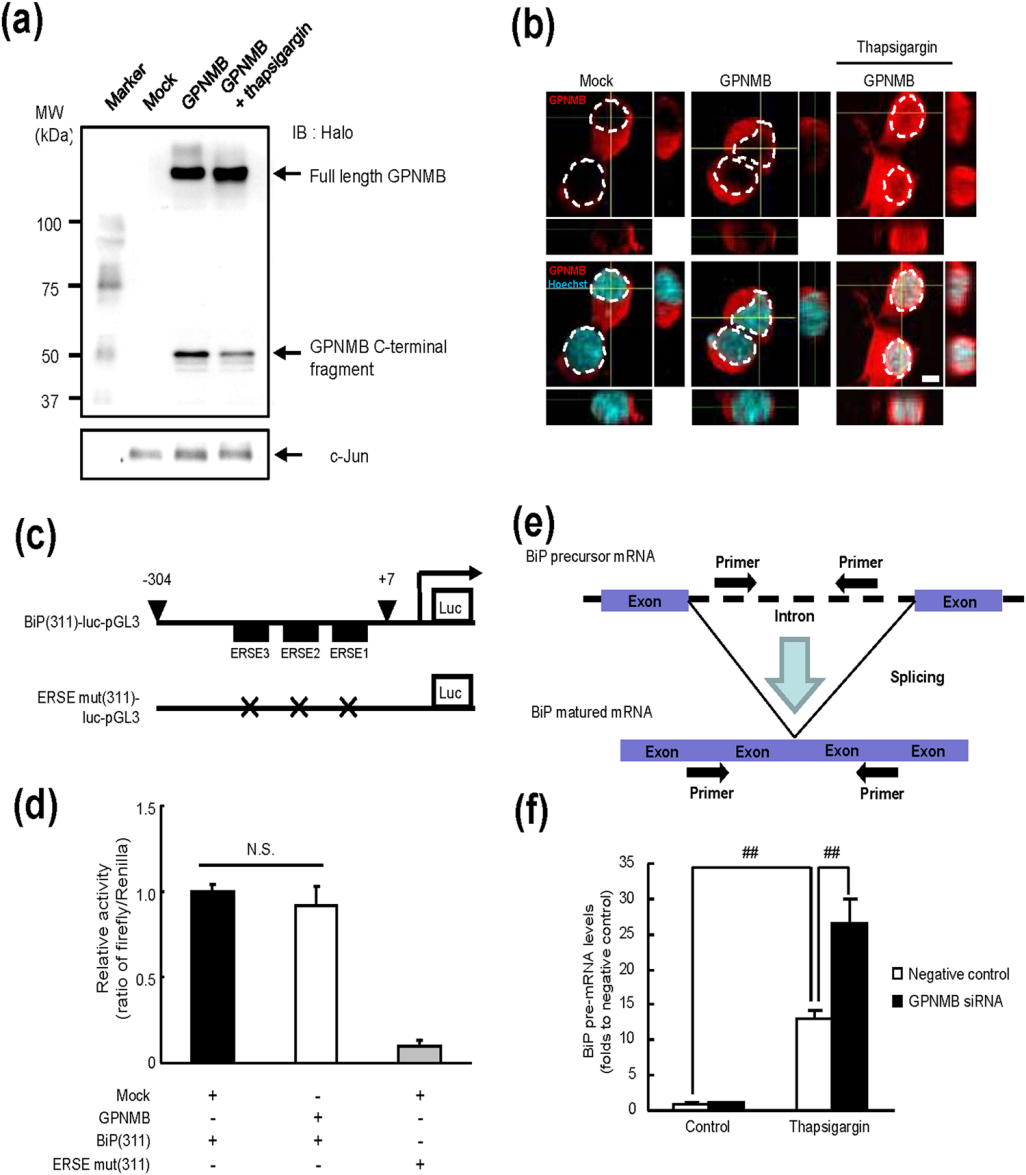
GPNMB relocates into the nucleus and enhances *BiP* mRNA splicing. (**a**) GPNMB expression in the nuclear fraction in the NSC34 cells transfected with the mock or GPNMB-Halo tag vector or the GPNMB-Halo tag vector with thapsigargin treatment at 1 μM. (**b**) By means of incubation with 1 μM thapsigargin, nuclear translocation of GPNMB was detected in NSC34 cells transfected with the GPNMB-Halo tag vector (red; GPNMB, blue; Hoechst 33342). The scale bar is 5 μm. (**c**) Schematic representation of the *BiP* promoter that was cloned into the pGL3 plasmid (BiP[311]-luc-pGL3) and of the ERSE mutant *BiP* promoter that was cloned into the pGL3 plasmid (ERSE mut[311]-luc-pGL3). (**d**) The relative reporter activity in cells transfected with enhanced green fluorescent protein (GFP)-tagged mock plasmid or GPNMB-Halo tag, BiP(311)-luc-pGL3, and/or ERSE mut(311)-luc-pGL3 vectors 12 h after incubation with 1 μM thapsigargin. The data are presented as mean ± SEM (*n* = 4). (**e**) Schematic representation of primers for the precursor and mature mRNA of murine *Gpnmb*. (**f**) Expression of *BiP* pre-mRNA was measured using RT-PCR. Cells transfected with either anti-*Gpnmb* siRNA or negative control siRNA were incubated with 1 μM thapsigargin for 12 h. The data are presented as mean ± SEM (*n* = 5). ^##^ *P* < 0.01 (Tukey’s test).

As shown in Figs 2b and 4, the BiP expression was enhanced specifically by GPNMB at the mRNA level under the conditions of ER stress without the involvement of the major ER stress response pathways. Thus, we focused on regulation of the *BiP* promoter activity and/or *BiP* mRNA maturation by GPNMB, and we first studied alterations of the intracellular location of GPNMB by ER stress. As shown in Fig. 5a, the amount of full-length GPNMB with the Halo tag (glycosylated GPNMB) was increased in the nucleus 12 h after the thapsigargin treatment, whereas the Halo-tagged GPNMB fragment with the molecular weight of approximately 50 kDa was down-regulated. The nuclear localisation of GPNMB under ER stress was also observed in the immunofluorescent images (Fig. 5b).

It is known that the mammalian ER stress-response element (ERSE) is present in the promoter regions of UPR’s target genes such as *BiP* ^30^. Therefore, we examined the effects of GPNMB on the expression of a reporter gene carrying the human *BiP* promoter (between positions −304 and +7), which contains three tandem ERSE sitesor mutant ERSE sites immediately upstream of the firefly luciferase gene (BiP[311]-luc-pGL3 and ERSE mut[311]-luc-pGL3; Fig. 5c), as described elsewhere^30^. The relative reporter activity in the cells transfected with mock and BiP(311)-luc-pGL3 vectors was increased by the thapsigargin-induced ER stress, whereas that in the cells transfected with ERSE mut(311)-luc-pGL3 was not changed (Fig. 5d). Furthermore, there were no changes in these activities between mock-transfected and GPNMB-overexpressing cells, both transfected with the BiP(311) vector (Fig. 5d), indicating that GPNMB is unlikely to control the *BiP* transcription activity in response to ER stress. To test whether GPNMB mediated splicing of *BiP* pre-mRNA, we next performed relative quantitative real-time PCR using specific primers recognizing the GPNMB intron region (targeting the 2,804–2,829 and 2,938–2,961 sites; Fig. 5e). Thapsigargin treatment of NSC34 cells that were transfected with the negative control siRNA increased the *BiP* pre-mRNA level (Fig. 5f).

The amount of *BiP* pre-mRNA after ER stress was increased, not reduced, by the knockdown of GPNMB, in comparison with the negative control group (Fig. 5f). This result was indicative of the lower transcriptional activity of *BiP* in the GPNMB knockdown cells, suggesting that GPNMB may act as an enhancer of *BiP* pre-mRNA splicing.

### *Gpnmb*-transgenic mice are protected from neuronal damage after cerebral ischemia

**Figure 6:**
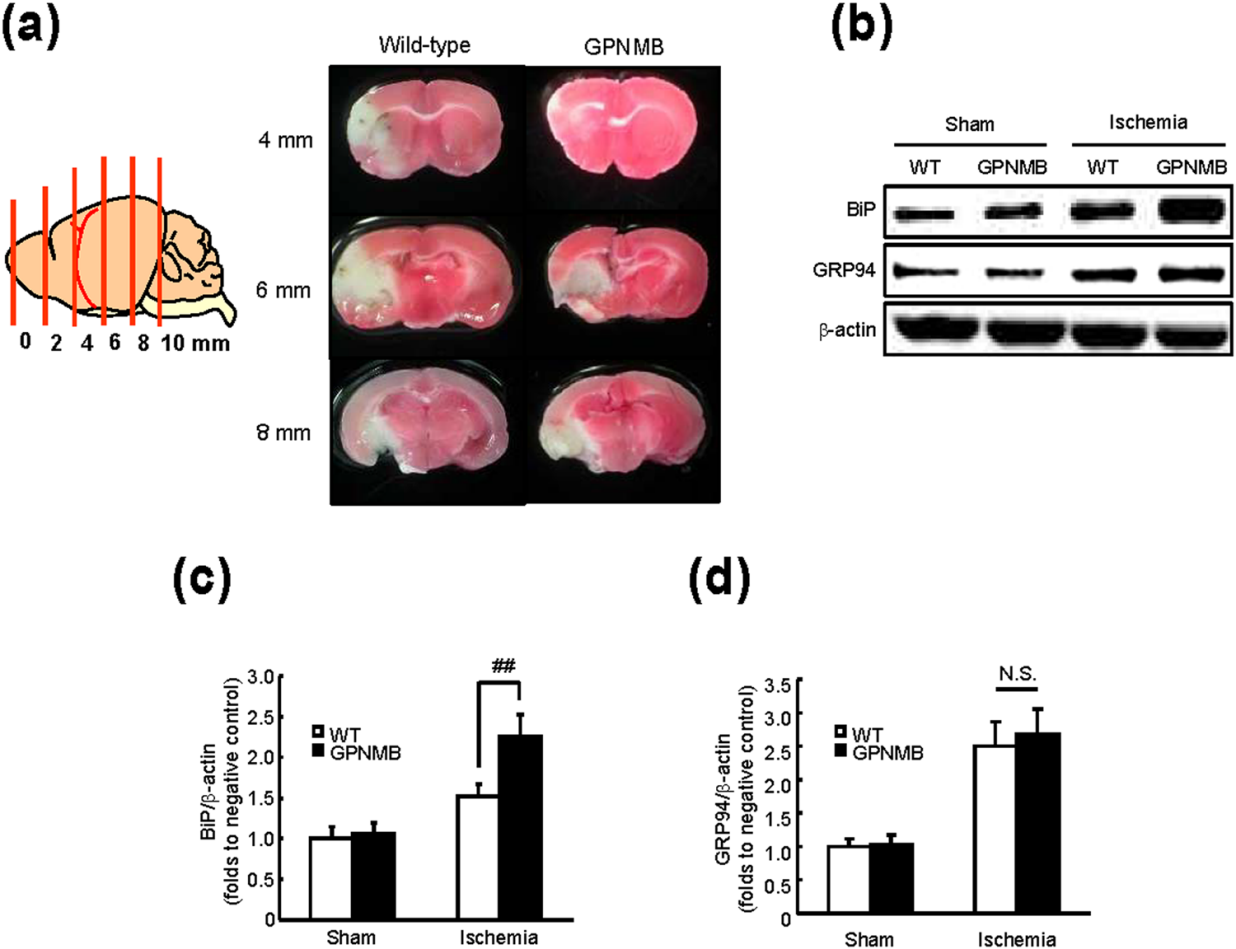
The up-regulation of BiP protein expression in *Gpnmb*-transgenic mice after middle cerebral artery occlusion (MCAO; 2 h of ischemia and 22 h of reperfusion). (**a**) Images of a brain slice and triphenyltetrazolium chloride (TTC) staining after MCAO. (**b**) The BiP and GRP94 expression levels in *Gpnmb*-transgenic and wild-type (WT) mice after MCAO or a sham operation. The protein levels of BiP (**c**), but not GRP94 (**d**), were increased in *Gpnmb*-transgenic mice after MCAO, in comparison with WT mice. The data are presented as mean ± SEM (*n* = 5 to 9). ^##^ *P* < 0.01 (Tukey’s test).

Finally, we confirmed the role of GPNMB in BiP expression using *Gpnmb*-transgenic mice after cerebral ischemia that was induced by the middle cerebral artery occlusion (MCAO). According to our previous reports, ER stress plays an important causal role in experimental models of permanent ischemia^17^ and ischemia/reperfusion^18^. The infarct volume in *Gpnmb*-transgenic mice after MCAO was decreased in comparison with wild type (﻿WT) mice (Fig. 6a). Indeed, we observed up-regulation of BiP and GRP94 after MCAO in WT mice (Fig. 6b–d). In *Gpnmb*-transgenic mice after MCAO, the protein level of BiP, but not of GRP94, was significantly increased (1.5-fold) in comparison with WT mice (Fig. 6b–d). Furthermore, there was no significant change in BiP between WT and *Gpnmb*-transgenic mice in the sham group, indicating that GPNMB acted as an ER stress responder and accelerated the *BiP* induction under the pathological conditions, i.e. during brain ischemia.

## Discussion

This seems to be the first report showing a relation between GPNMB and the ER stress response. Our present results suggest that GPNMB specifically induces expression of BiP through the direct regulation of pre-mRNA splicing but not *via* the major UPR pathways, thereby resulting in protection of the cell from ER stress. The high expression of BiP and the neuroprotection from ischemic damage in *Gpnmb*-transgenic mice after cerebral ischemia suggest that GPNMB is an important participant in the induction of BiP under pathological ischemic conditions. Here, we propose a theory that GPNMB is a novel ER stress regulator and is necessary for the BiP up-regulation, which protects the cell from death caused by ER stress. In the evaluation of UPR pathways (Fig. 4), we could not use other cell lines to confirm that GPNMB has no effects for UPR pathways. There are some reports which investigated the relationship GPNMB and ER-stress. In RAW264.7 which derived from murine macrophage, thapsigargin and tunicamycin were not induced GPNMB expression. Moreover, tunicamycin did not affect the cell survival of primary cultured osteoblasts^33^. These researches suggest that the reactivity of GPNMB against ER-stress may be different in each cell types. Therefore, we need to do further investigation.

The expression level of the GPNMB protein is immediately increased by thapsigargin treatment without up-regulation of *Gpnmb* mRNA (data not shown). The GPNMB protein can be ubiquitinated^19^; therefore, the expression level of GPNMB may be regulated by a degradation system such as the ubiquitin-proteasome system, and GPNMB may respond not only to ER stress but also to some other stressors such as hypoxia. The knockdown and overexpression of GPNMB do not affect the BiP expression under normal conditions (Fig. 2b and c), indicating that GPNMB requires some modifications that alter translocation and/or cofactors that are activated by cellular stress.

Splicing of pre-mRNA involves elimination of introns during the gene transcription and ligation of the exons that form the mRNA. This process is mediated by the recognition of specific sequences at the exon-intron boundaries by splicing factors such as small nuclear ribonucleoprotein particles (snRNPs) and serine/arginine-rich proteins (SR proteins)^34–36^. After sensing unfolded proteins, IRE1 oligomerises and is activated by auto-phosphorylation; then it initiates the non-conventional splicing of *HAC1* mRNA a transcription factor in yeast or its mammalian homologue, *XBP1*, by cleaving the mRNA at two conserved sites to excise an intron^37, 38^. Spliced *XBP1* mRNA is translocated to the nucleus as a potent transcription factor that activates the transcription of *BiP* by binding to ERSE^31, 39^. Contrary to our expectations, our results show that GPNMB regulates the *BiP* pre-mRNA splicing without activation of the IRE1 pathway. These findings point to the possibility of regulation of the *BiP* pre-mRNA splicing by GPNMB: (i) GPNMB may work as a splicing factor itself, leading to direct control of the *BiP* pre-mRNA splicing, or (ii) GPNMB may stimulate the activity of other splicing factors related to the BiP induction; for example, by interacting with snRNPs or SR proteins, or conversely, by interfering with the action of splicing inhibitors such as the heterogeneous nuclear ribonucleoproteins (hnRNPs). A recent study revealed that the C-terminal fragment of GPNMB interacts with hnRNP A1 and the splicing factor arginine/serine-rich 9 (SFRS9)^40^. Thus, GPNMB may be a cofactor of hnRNP A1 and/or SFRS9 and may drive the *BiP* pre-mRNA splicing *via* interaction with these factors. Full-length GPNMB is up-regulated in the nucleus (Fig. 5a and b) under ER stress, whereas the C-terminal fragment of GPNMB is down-regulated there (Fig. 5a). Moreover, overexpression of the GPNMB C-terminal fragment does not affect the BiP expression, although the fragment can be translocated to the nucleus (data not shown). Therefore, the full-length GPNMB is necessary to increase the BiP expression, whereas the C-terminal region of GPNMB may be necessary for binding to the splicing factors. In this study, we demonstrated that GPNMB did not regulate ERSE activity. However, we could not investigate the interaction between GPNMB and other promoters. We need to clarify other pre - mRNAs which affected by GPNMB because there is a possibility that GPNMB interacts other pre-mRNAs which have cytoprotective effects. Moreover, to evaluate the splicing effect of GPNMB directly, it is required to use other methods such as *in vitro* splicing assay.

ER stress is implicated in many neurodegenerative and familial protein folding disorders as well as in several cancers and a number of inflammatory diseases including diabetes, atherosclerosis, inflammatory bowel disease, skeletal muscle diseases, and arthritis^41^. For example, recent studies clearly showed that ER stress and the UPR are robustly up-regulated in various tumour cell types and are closely associated with cancer cell survival and resistance to anti-cancer treatments^19^. During the abnormal proliferation of cancer cells, the ER is overloaded and requires increased activities of protein folding, assembly, and transport, leading to physiological ER stress^42^.

As for BiP, it was shown to be up-regulated in several types of cancer, e.g. hepatocellular carcinoma, renal carcinoma, and breast cancer^43^, and to be involved in cancer pathophysiology by conferring chemoresistance to tumour-associated endothelial cells^44^. Indeed, aberrant GPNMB expression has been linked to several cancers such as glioma^45^, hepatocellular carcinoma^46^, cutaneous melanoma^47^, and breast cancer^48^. Thus, these findings suggest that the high levels of GPNMB expression in cancer cells are maintained for the purpose of BiP induction, which has a strong cytoprotective effect during oncogenesis^49^. In skeletal muscle, where GPNMB regulates degeneration/regeneration of the extracellular matrix^50^, ER stress was implicated in the pathogenesis of autoimmune muscle diseases, such as polymyositis and dermatomyositis, because of enhancement of the expression of BiP and other ER stress-related gene^51^. Moreover, we demonstrated BiP induction in *Gpnmb*-transgenic mice after cerebral ischemia in the present study. In the recent research, the original function of GPNMB was elucidated that it interacts with calnexin, a kind of molecular chaperon and reducing oxidative stress in hepatocytes and stellate cells^52^. These findings suggest that the functions of GPNMB are specific according to cell types. Thus, from the therapeutic perspective, elucidation of the role of GPNMB in stress responses may lead to better treatments of several disorders, including stroke, neurodegenerative and muscle diseases, and several cancers.

In conclusion, we demonstrated that GPNMB protects neuronal cells from death caused by ER stress because this protein promotes splicing of *BiP* pre-mRNA. This finding may facilitate the development of new therapies for some ER stress-related disorders.

### Experimental procedures

#### Plasmids

Human GPNMB cDNA containing the entire coding region was purchased from Kazusa DNA Research Institute (Chiba, Japan). A pGL3-BiP promoter (311)-Luciferase reporter plasmid (BiP [311]-pGL3) and an ER stress response element (ERSE) mutant BiP promoter-luciferase reporter plasmid (ERSE mut [311]-pGL3) were provided by Prof. Kazutoshi Mori (Kyoto University, Kyoto, Japan).

### Cell culture and transfection

Murine motor neuron (NSC34) cells were purchased from Cosmo Bio Co., Ltd. (Tokyo, Japan) and maintained in Dulbecco’s modified Eagle’s medium containing with 10% foetal bovine serum (Valeant, Costa Mesa, CA, USA), 100 U/mL penicillin (Meiji Co., Ltd., Tokyo, Japan), and 100 µg/mL streptomycin (Meiji) in a humidified atmosphere of 95% air and 5% CO_2_ at 37 °C. The cells were passaged by trypsinisation every three to four days.

HT22 cells which derived from mouse hippocampal cells were a generous gift from Yoko Hirata Ph.D. (Gifu University, Gifu, Japan). In the experiment of Fig. 2f and g, HT22 cells were seeded to 24 well plates at 15,000 or 3,000 cells per wells and cells were incubated with thapsigargin at 50 nM. HT-22 cells were maintained in the same method of NSC-34 cells. Transfection with an empty vector (mock control), full-length GPNMB C-terminal Halo tag, or pGL3-BiP promoter plasmids was performed using Lipofectamine 2000 (Thermo fisher scientific, Waltham, MA, USA) following the manufacturer’s instructions. Full-length GPNMB C-terminal Halo tag vector was originally synthesized from N-terminal Halo tag vector (pFN21AB9985: kazusa DNA Res. Inst., Chiba, Japan) and p-FC14K HaloTag^®^ CMV Flexi^®^ Vector (kazusa DNA Res. Inst.).

### The knockdown of GPNMB by means of small interfering RNA (siRNA)

NSC34 cells and HT22 cells were transfected with 100 nM of a mouse anti-*Gpnmb* RNAi oligo and a control RNAi oligo (Nippon EGT Co., Ltd., Toyama, Japan) using Lipofectamine RNAiMAX (Thermo fisher scientific) according to the manufacturer’s instructions. The knockdown was verified by immunoblotting with an antibody to GPNMB. The siRNA sequences were as follows: 5′-GCACGGGUUUCUAUAAACAdTdT-3′ (sense) and 5′-UGUUUAUAGAAACCCGUGCdTdT-3′ (antisense) and a universal negative control siRNA.

### The cell death assay

NSC34 cells were seeded (10,000 per well) in a 96-well plate and then incubated at 37 °C in a humidified atmosphere of 95% air and 5% CO_2_. After 24 h, the cells were transfected with a negative controlor GPNMB siRNA using Lipofectamine RNAiMAX or with the mock or GPNMB-Halo tag vector using Lipofectamine 2000 for 48 h, and were then treated with thapsigargin (1 μM; Wako Pure Chemicals, Osaka, Japan), which is an ER stress inducer, for 27 h. In the experiment of Fig. 3a, the cells were incubated with thapsigargin and a recombinant extracellular fragment of human GPNMB (Bio-techne, Minneapolis, MN, USA) at the final concentration of 0.025, 0.25, or 2.5 μg/mL. Cell death was quantified on the basis of combination staining with fluorescent dyes (namely, Hoechst 33342 and propidium iodide [Thermo fisher scientific]), with examination under an OLYMPUS IX70 inverted epifluorescence microscope (Olympus, Tokyo, Japan). In a blinded manner, at least 200 cells per condition were counted using image-processing software (ImageJ, version 1.33 f; National Institutes of Health, MD, USA).

### RNA isolation

Total RNA was purified from the NSC34 cells using the Qiagen RNeasy Mini Kit (Qiagen, Valencia, CA, USA) according to the manufacturer’s instructions. RNA quantity and quality were determined using NanoVue Plus (GE Healthcare UK, Ltd., Buckinghamshire, England).

### Real-time PCR

Single-stranded cDNA was synthesised using the PrimeScript RT Master Mix (Takara, Otsu, Japan). Species-specific primer sets were designed: for mouse *BiP* mRNA, 5′-CTCCACGGCTTCCGATAATCA-3′ (sense) and 5′-TCCAGTCAGATCAAATGTACCCAGA-3′ (antisense); for mouse *Grp94* mRNA, 5′-TTTGAACCTCTGCTCAACTGGATG-3′ (sense) and 5′-CTGACTGGCCACAAGAGCACA-3′ (antisense); for mouse calreticulin mRNA, 5′-TTTCCGAGTGGTTTGGACCAG-3′ (sense) and 5′-GATCAGCACATTCTTGCCCTTG-3′ (antisense); for mouse pre-*BiP* mRNA, 5′-TCAGGCTTAGTTAAGATCGGTAGCA-3′ (sense) and 5′-GTCCTGAGAGCACTACCAAGCAA-3′ (antisense); and for mouse *Xbp-1* mRNA 5′-GAACCAGGAGTTAAGAACACG-3′ (sense) and 5′-AGGCAACAGTGTCAGAGTCC-3′ (antisense). Glyceraldehyde 3-phosphate dehydrogenase (*GAPDH*) mRNA served as a loading control with the primers 5′-TGTGTCCGTCGTGGATCTGA-3′ (sense) and 5′-TTGCTGTTGAAGTCGCAGGAG-3′ (antisense).

Relative quantitative real-time PCR was performed using the Thermal Cycler Dice Real Time System TP800 with SYBR Premix Ex Taq II (Takara), according to the manufacturer’s protocols. The thermal cycling conditions were as follows: 5 s at 95 °C and then 30 s at 60 °C, followed by two-step PCR for 50 cycles consisting of 95 °C for 15 s and 60 °C for 1 min. For each PCR, we obtained a slope value, R2 value, and the linear range of the standard curve of serial dilutions. The results were expressed relative to the *GAPDH* internal control.

### Immunoblot analysis

Cells and brain tissues were lysed in a special buffer (RIPA buffer R0278; Sigma-Aldrich, St. Louis, MO, USA) with a protease inhibitor cocktail (cat. # P8340; Sigma-Aldrich) and phosphatase inhibitor cocktails (cat. ## P2850 and P5726; Sigma-Aldrich). The protein concentration was measured *via* comparison with a known concentration of bovine serum albumin using the BCA Protein Assay Kit (Thermo Fisher Scientific). Equal amounts of protein in sample buffer containing 10% 2-mercaptoethanol were subjected to sodium dodecyl sulphate polyacrylamide gel electrophoresis (SDS-PAGE) in 5–20% gradient gels (SuperSep Ace; Wako), and the separated proteins were transferred onto a polyvinylidene difluoride membrane (Immobilon-P; Merck Millipore Corporation, Bedford, MA, USA). After blocking with Blocking One-P (Nacalai Tesque), we incubated the membranes with the following primary antibodies: a goat anti-GPNMB (Bio-techne), mouse anti-BiP (BD Bioscience, San Jose, CA, U.S.A.), rabbit anti-phosphorylated eukaryotic initiation factor 2 α (eIF2α; Cell Signaling Technology, Beverly, MA, USA), rabbit anti-eIF2α (Cell Signaling Technology), rabbit anti-activating transcription factor 6 (ATF6; Abcam, Cambridge, MA, USA), and a mouse anti-β-actin antibody (Sigma-Aldrich). After that, the membrane was incubated with the following secondary antibodies: a horseradish peroxidase-conjugated rabbit anti-goat IgG (Thermo Fisher Scientific), goat anti-rabbit IgG, or a goat anti-mouse IgG antibody (Thermo Fisher Scientific). The band intensity was measured using an Immuno Star LD (Wako). Band intensity was measured using an LAS-4000 UV mini Luminescent Image Analyzer (Fujifilm, Tokyo Japan) and Multi Gauge Version 3.0 (Fujifilm). For quantitative analysis, total protein signals were used as controls for phosphoprotein signals. Equal loading was confirmed using β-actin and total protein signals were confirmed as controls for phosphoprotein signals.

### Nuclear extracts

They were prepared by means of the Subcellular Proteome Extraction Kit (S-PEK; Merck Millipore Corporation) according to the manufacturer’s instructions. Briefly, NSC34 cells were plated in 10-cm dishes at 2.0 × 10^7^ cells per dish and were transfected with a Myc-tagged mock plasmid or GPNMB-Halo tag plasmid for 48 h. After 27 h incubation with or without thapsigargin, the cells were harvested by trypsinisation and centrifuged at 100 × *g* for 10 min to obtain the cell pellets. The pellets were resuspended in cold wash buffer, incubated for 5 min on ice, and then centrifuged at 100 × *g* for 10 min. The pellets were resuspended in 100 μL of extraction buffer 1 (containing 0.5 μL of the protease inhibitor cocktail [Sigma-Aldrich]) and were incubated for 10 min on ice. Then, the mixture was centrifuged at 1,000 × *g* for 10 min, and the supernatant was discarded. Next, the insoluble pellets were resuspended in 100 μL of extraction buffer 2 (containing 0.5 μL of the protease inhibitor cocktail [Sigma-Aldrich]) and were incubated for 10 min on ice. The mixture was centrifuged at 6,000 × *g* for 10 min, and the supernatant was discarded. Finally, the insoluble pellets were resuspended in 100 μL of extraction buffer 3 (containing 0.5 μL of the protease inhibitor cocktail [Sigma-Aldrich] and 0.15 μL of benzonase nuclease) and were incubated for 10 min on ice. After that, the mixture was centrifuged at 6,000 × *g* for 10 min, and the supernatant was collected. The supernatants were stored in aliquots at −70 °C.

### The luciferase assay

This assay was performed by means of a Dual-Luciferase Reporter Assay System (Promega, Madison, WI, USA) as described previously^27^. Briefly, NSC34 cells were transfected with GFP-tagged Mock and BiP (311)-pGL3, GPNMB-Halo tag and BiP (311)-pGL3, or GFP-tagged Mock and ERSE mut (311)-pGL3 (Promega). At 48 h after the transfection, the cells were incubated with 1 μM thapsigargin for 12 h. Firefly and *Renilla* luciferase activities were measured in 20 μL of a cell lysate using a Dual-Luciferase Reporter Assay System (Promega) and a spectrophotometer (Varioskan; Thermo Fisher Scientific). Relative luciferase activity was defined as the ratio of the firefly luciferase activity to *Renilla* luciferase activity.

### Mice

The experimental designs and all procedures were in accordance with the guidelines of the World Medical Association’s Declaration of Helsinki and the U.S. Department of Health and Human Services Guide for the Care and Use of Laboratory Animals and the study was approved by the Experimental Committee of Gifu Pharmaceutical University. Mice were randomized and operators were blinded. Transgenic mice overexpressing GPNMB were provided to Gifu Pharmaceutical Universityby the University of Tokushima Graduate School (Prof. Takeshi Nikawa). All mice were housed in a room under the lighting conditions of 12 h light and 12 h dark.

### Transient focal cerebral ischemia

An experimental model of focal cerebral ischemia was set up as described previously^53, 54^. Briefly, the mice were anaesthetised *via* inhalation of 1.0–1.5% isoflurane (Mylan, Inc., Canonsburg, PA, USA) with air (21% O_2_) by means of a face mask. Focal cerebral ischemia was induced by introducing an 8–0 monofilament (Ethicon, Somerville, NJ, USA) coated with a silicone hardener mixture (Xantopren; Bayer Dental, Osaka, Japan) into the left common carotid artery and by advancing the monofilament along the internal carotid artery until the tip occluded the proximal stem of the middle cerebral artery (MCA). Two hours after the ischemia, the animals were briefly reanaesthetised with isoflurane, and the filament was withdrawn to allow for reperfusion through the common carotid artery. During the surgical procedure, body temperature was maintained between 37.0 °C and 37.5 °C by means of a heating lamp and heating pad. Mice with an intracranial haemorrhage, pulmonary insufficiency, and/or asphyxia and/or without an ischemic brain infarct were excluded from subsequent analysis. At 18 h after the reperfusion, 2-mm-thick brain slices were prepared and immersed for 20 min in a 2% solution of 2-, 3-, or 5-triphenyltetrazolium chloride (TTC; Sigma-Aldrich). The infarction area was photographed with a digital camera (Coolpix 4500; Nikon, Tokyo, Japan) and measured using image-processing software (ImageJ version 1.33 f). The volume of infarction was calculated by numeric integration of infarct areas × section thickness as described previously^53^.

### Data analysis

The data are presented as mean ± SEM. Statistical comparisons were made by Student’s *t*-test, one-way analysis of variance (ANOVA) followed by Dunnett’s test, and Tukey’s test in the STAT VIEW software, version 5.0 (SAS Institute, Cary, NC, USA). A *P* value < 0.05 was assumed to indicate statistical significance.
